# Positive intervention for depression and teacher–student relationship in Iranian high school girl students with moderate/mild depression: a pilot randomized controlled trial

**DOI:** 10.1186/s13034-020-00331-9

**Published:** 2020-06-03

**Authors:** Ali Taghvaienia, Arash Zonobitabar

**Affiliations:** Department of Education and Psychology, Faculty of Humanities, Yasouj University, Yasouj, Iran

**Keywords:** Positive intervention, Depression, Teacher–student relationship, Students, High school

## Abstract

**Background:**

Positive intervention (PI) is a modern and therapeutic approach broadly based on the principles of positive psychology (Rashid in J Posit Psychol 1:25–40, 2014). PI effects at schools have received little attention to date. However, since PI offers a focus on the positive aspects of human experience (Seligman and Csikszentmihalyi in Am Psychol 55:5–14, 2000), we hypothesized that it could exert positive changes in the teacher-student relationship (TSR) and depression symptoms. Therefore, the mentioned pilot study conducted in this article aimed at evaluating PI effects on depression and TSR among Iranian high school girl students with moderate/mild depression.

**Method:**

In this research, 60 eligible female students (aged 15–18) with a Beck Depression Inventory-II (BDI-II) mild-moderate depression diagnosis, were randomly divided into PI (*n *= 30) and control groups (*n *= 30) at the time of entering the study and 2 months later following their assessments through the Inventory of TSR (IT-SR) and BDI-II. The intervention group participated in 8-week 2 h group sessions of PI and the control group was evaluated without any intervention.

**Results:**

The study was completed with a total number of 49 girls [PI group (*n *= 24), and control group (*n *= 25)] and everybody participate in 8 sessions. The intervention group was effective on the variables of BDI-II and IT-SR in a way that the involved girls increased their communication (*p *= 0.001, *d *= 0.17), trust (*p *= 0.001 *d *= 0.14) after PI training and decreased alienation (*p *= 0.012, *d *= 0.11) and depression (*p *= 0.001, *d *= 0.15) among other high school students.

**Conclusion:**

This intervention could function as an unspecific component of a stepped care approach for teenage girls suffering from depression. This study recommends more RCT with large sample sizes among high school boys students and follow-up.

## Background

Depression is one of the most common mental illnesses among high school students [24]. A study in Iran has shown that nearly 34% of high school students experience depression and 25% of male and 39% of female students have depressive symptoms [40]. Normal stress of adolescence has been commonly known to be the cause of depressive symptoms among high school students [21]. Its symptoms have been misdiagnosed for primary conduct and attentional problems and substance abuse or else seen as the youth’s going-through stage [48]. Suicide rates increased (37%) from 1999 through 2014 (from 1.9 to 2.6) for females aged 10–14 and an increased suicide risk has been reported to be induced by depression [14]. In addition to their enhanced suicide risks, depressed Iranian students are involved in higher risks of mental disorders, including conduct disorders and stress, sexual abuse as well as using alcohol or other drugs [18] and it is more probable that they engage in unsafe sexual intercourses or any other risk behaviors as compared to other students [59]. Moreover, such students may experience difficult relationships with their teachers. However, this problem has not been well understood [46].

Therefore, now researchers are trying to focus their attention on teacher–student relationship (TSR) development [7]. This diversely emerging field of study has theoretically included the positive and negative dimensions of emotional support and conflict followed by involvement and alienation in the definitions of students’ supportive and non-supportive relationships with their teachers, respectively [41]. TSR quality is one of the critical factors within the school environment that predicts high school students’ immediate and future outcomes including students’ academic achievement, affect, behavior, and motivation [28, 56, 57]. However, depressed students perceive their teachers as less supportive, and show more academic failure [20, 55]. It’s important to address TSR in treating adolescent depression, because evidence has shown that quality of interpersonal relationships can significantly predict depression [39] and interpersonal relationships plays a significant role to prevent and overcome adolescent depression [25].

The research in this field is indicative of the presence of less personal and more formal, evaluative, and competitive relations between teachers and high school students with depression [35]. So, these challenges can lead to undesirable self-evaluations and negative attitudes towards classroom learning [56] because the evaluative and non-personal natures of TSR qualities in high schools do not correspond to the depressed students’ communicative needs [45]. Hence, there is a need for some supportive and professionally appropriate interventions in hope of finding some ways to counteract some of the factors that may destroy the relationships between teachers and students and adversely affect the qualities of educations provided for students in turn [12]. Furthermore, even if TSR are causally associated with only a fraction of the student benefits they are correlated with, successful interventions for improve TSR and reducing depression symptoms would be a excellent boon to any high school [7].

Students’ depressive symptoms have been attempted to be relieved through various interventions, such as cognitive behavioral therapy, cognitive therapy based on self-help mindfulness treatment, qigong movement therapy, electroacupuncture, and regular exercise [6, 9, 16, 26, 34]. Nevertheless, these standard methods have been associated with a variety of challenges. Although most traditional therapies have been interesting for depressed people, they have aimed at improving personal deficits by focusing on only depressive symptoms [22]. Furthermore, these therapies exclusively stressing treatments of deficient thoughts and behaviors and some people may withdraw during their treatments. Most importantly, students’ mental health might be improved by only few interventions as their personal assets and strengths are appropriately targeted [11]. Therefore, their outcomes may merely include disappearing of only depressive symptoms, while little emphasis is placed on positive attributes. These symptoms have been seriously lagging behind amelioration of students’ positive emotions and relationships with their teachers, constructive behaviors, and social bonds in schools [32, 49].

Positive intervention (PI) was initially developed by Seligman, Rashid, & Parks, for depression as a target condition in need of intervention following the hypothesis that depression can be treated not only by effectively reducing its negative symptoms, but also by primarily and directly building positive emotions, feelings, character strengths, engagement, and meaningfulness in the involved patients [52]. PI is a modern, structured, and therapeutic approach broadly based on the principles of positive psychology [44]. It is intended to induce and maximize positive effects by minimizing them and enhance subjective well-being and happiness by elevating optimism and gratefulness [43]. Seligman [53] described the 3 elements of positive emotion, engagement, and meaning for ‘happiness’ as the ultimate goal of positive psychology. Positive emotions, including optimism, confidence, trust, hope, satisfaction, and pride, counteract negative and detrimental emotions in physiology [19]. Engagement is defined as construction of an engaged life via involvement and assimilation at leisure and work times, as well as in close relationships [13]. Meaning is related to pursuing a meaningful life by pertaining and serving anything greater than oneself when one is encouraged to use his/her talents and strengths of character [52]. PI seeks to alleviate them as the causes of depression by primarily and directly using students’ personal assets and strengths and building their positive emotions, engagements, and meanings instead of targeting only depressive symptoms, as well as, due to having a focus on the positive aspects of human experience [15, 51], PI was theorized to induce some positive changes in TSR in this research. Studies have shown that PI can promote interpersonal relationships, and communication skills in students [32]. One study revealed that positive Iranian-Islamic therapy had a greater impact on the reduction of depression of high school girl students with social anxiety compared to positive existential therapy [38]. According to the results of four recent meta-analyses [5, 8, 23, 54] PI dicreases depression symptoms. Nevertheless, the studies that entered the meta-analyses also highlighted one group that was not dealt with. Surprisingly, exploring the one meta-analyses revealed that only 3 out of the 74 studies focused on high school students with depression symptoms (i.e., used samples with a mean age under 18 years). In addition, it was found that these studies were RCTs with common control groups (untreated groups) and small effect sizes. Exploring the one of the studies that entered the meta-analyses showed that positive group psychotherapy helped improve self-esteem, interpersonal relationships, and communication skills in 33 students [32]. Exploring the mean age of the participants in the studies that entered the meta-analyses revealed that majority of them focused on older participants–frequently college students. Therefore, information on the applicability and efficiency of such interventions in samples aged 18 and younger is quite incomplete.

To date, no comprehensive studies have been done to assess the effect of PI training on TSR among high school students. In addition, we do not have enough data about the standard treatment protocol with PI for high school students in terms of TSR and depression symptoms and a pilot randomized controlled trial should be conducted to nominate PI for an improved TSR and reduced depression. Therefore, the effects of PI on depression and TSR of Iranian high school girl students with moderate/mild depression, was investigated using a pilot randomized controlled trial. So, we hypothesise that participants receiving the intervention will experience, compared to the control group, an improvement in depression symptoms and TSR.

## Methods

### Study design and subjects

To investigate PI effects on the depression and TSR of Iranian high school girl students with moderate/mild depression in this pilot randomized controlled trial, first, 170 students studying in the girls high schools of Fars Province (IR Iran) were selected through multi-stage cluster sampling by a researcher. To this goal, from the five educational districts of Fars Province, 2 districts were randomly selected, 2 schools were randomly chosen from each district, and from each school 3 classes were randomly allocated for test. The students in these 6 classes completed the BDI-II. 60 students diagnosed with a BDI-II mild-moderate depression were considered as a statistical sample in each group, and were distributed into the two PI (*n *= 30) and (*n *= 30) control groups by random (via an automated random number generator).

Before participating in the study, the students were evaluated for eligibility by a school consultant. The inclusion criteria were as follows: (a) being a female of 15–18 years of age, (b) having a score on the BDI-II between 14 and 28 (mild-moderate depression), (c) having passed at least 1 term of education at high school, (d) not practicing PI before participating in this study, (e) not taking daily medications for depression symptoms, as well as (f) no showing evidence of mental disorders, such as conduct disorder, panic disorder, obsessive–compulsive disorder, anorexia nervosa, bulimia nervosa, borderline personality disorder or other mental disorders, such as substance-related and addictive disorders according to the participants’ parents, or peers, and Diagnostic and Statistical Manual of mental disorders (DSM-5 criteria) [1], that could make an interference in TSR and/or depression. Girl students were selected as a specific group, because evidence has shown that the incidence of depression among high school students is greater females than males [58]. Due to the ethical issues of choosing female students, researchers intend to conduct a similar study on high school male students with moderate/mild depression. Data were collected by a researcher in the classroom.

### Intervention

#### Positive intervention (PI)

PI training was delivered in 8-week 2 h group sessions group sessions, which developed by Lee [32]; Table 1). There are previous studies utilizing this intervention among high school students (see [2, 32]. Positive psychology formed the basis of the concepts and theoretical grounds for PI [30]. The PI contents, including past satisfactions, present happiness, and future hope and optimism was chronologically ordered. Happiness was defined as a state well-being associated with flow (engagement in challenges with the help of one’s strengths and talents), a positive character displaying strength, talent, and interest, and a meaningful and purposeful life [30]. Students in the PI group were trained by a certified PI training coach, who had practiced PI for more than 2 years with experience in attending PI retreats. PI training coaches besides the 2 years of training are psychologists and therapists. Group sessions was conducted after school in the classroom. All participants can be in one group and one treatment providers deliver one group session. PI content was as follows: session 1 helped the selected high school students realize the meanings of their past and present pains. By applying PI in sessions 2–3, they understood their feelings and emotions somewhat better and discovered their strengths and negative aspects, which could lead to positive emotions and enhance their depressed moods. Dockray and Steptoe [17] showed that feelings and emotions were firmly associated with positive health outcomes. During the 4th session, the discoveries and resolutions of their family conflicts led to positive activities. Through session 5, they were helped to share their unhappy emotional experiences and reinterpret them. Furthermore, practicing gratitude and forgiveness by role-playing helped them to develop positive behaviors. Evidence has shown that positive activities elevate positive behaviors and thus reduce depression [37] and practicing gratitude is a protective factor against depression symptoms [29]. Sessions 6–7 focused on communication-skill training by role-playing and writing, which could more effectively improve their relationships and help them realize the meanings of life and death. These exercises could alleviate their negative depressive symptoms. Lyubomirsky et al. [36] found that appropriate communication could arouse subjective feelings of ‘meaning’ and good relationships would lead to people’s psychological well-being. Finally, session 8 focused on planning for the future. The students were asked to develop a detailed plan for their futures. This was useful for ensuring long-term effects of PI, including maintenance of the improved depressed moods.

**Table 1:** PI program overview

Session number	Name	Goals	Examples of activity
1	A curve of life	Introduce each individual, explore the meanings of pain and a purpose in life by understanding your past	Draw a curve of life after finding the meanings of the past and present pains
2	My present	Understand and know yourself	Talk about teacher–student relationship and write 10 sentences that show your understandings of feelings and emotions
3	A tree of life	Discover the positive and negative aspects of yourself	Draw a tree with good and bad fruits on it and talk about those you wish to keep or throw away
4	Family story	Discover your positive traits by helping to solve others’ family conflicts	Talk about the family members’ specifications of the past three generations and the conflicts between them after drawing their family tree with lines representing their closeness and problems, Exchange suggestions on resolving problems
5	Healing of the hurt mind	Seek health by understanding the concept of forgiveness	Share some experiences of the situations, in which you have received emotional scars and have had to forgive and be forgiven by role playing after writing a list of them
6	Good communication	Practice an appropriate communication	Role play in a recent stressful situation after writing about it or writing it through I-messages
7	Life and death	Practice coping with the concept of life and death, practice an appropriate communication with others	What would you write on your tombstone when you imagine your grave? Express expectations and needs as well as appreciations to your family and write apologies for the emotional wounds you have left behind during your life, while making your last demands
8	Planning the future	Accept responsibility for life	Know your wishes in life and make detailed plans

### Measures

#### Beck Depression Inventory- II (BDI-II)

In this study, the Beck Depression Inventory (BDI-II) was used to measure the severity of depressive symptoms. It is a 21-item scale and each item was scored from 0 to 3 (absent, mild, moderate and severe), according to the symptom severity where the total score ranged from 0 to 63. Higher scores represent more severe depression symptoms. The patient was diagnosed as depressed if they had a score less than 26, and the cut-off points used to determine depression levels were as follows: (a) normal (BDI-II score ≤ 13), (b) mild (14–19), (c) moderate depression (20–28), and (d) severe depression (29–63) [4, 33]. The BDI-II had a positive correlation with the Hamiltonian Depression Scale (*r *= 0.71), and its test–retest reliability was high (*r *= 0.93) indicating its good reliability [4]. The Cronbach’s alpha for the BDI-II total score was estimated 0.89 among adolescents [31]. The reliability of this study was estimated by using the Cronbach’s alpha coefficient of 0.79 for BDI-II.

#### Inventory of teacher–student relationships (IT-SR)

The study TSR was based on an IT-SR measure reported by a student and developed for the inventory of parent and peer attachment (IPPA; [3] widely utilized in the TSR context. Actually, the original IPPA has been designed to evaluate adolescents’ attachments to their parents and peers, to which 28 and 25 items are broadly allocated, respectively. In each section of the mentioned relationships, the 3 parallel factors of communication, trust, and alienation are included. In this research, 19 items adopted from the original IPPA were adapted for use in the TSR context to create the IT-SR. As recommended by Murray and Zvoch [42], 8, 5, and 6 items were loaded on the mentioned factors in the TSR questionnaires, respectively. A 4-point scale was applied to the provided responses and higher scores indicated higher TSR. Examples of items include: how much do you enjoy learning from <teacher’s name> ? How unfair is <teacher’s name> to you in class?). The IT-SR validity was considered to be acceptable for the research objectives as supported by some studies [41]. In this study, the mentioned factors were estimated as reliable via the Cronbach’s alpha coefficients of 0.78, 0.82, and 0.76, respectively.

### Outcome assessment

This type of BDI-II and IT-SR were employed to evaluate the members of the different study groups for depression and TSR at the pre-test and the post-test. The intervention group participated in 8 group sessions of PI and the control group was evaluated without any intervention. Assessments were collected by paper measure. A researcher explained the purpose and procedures of the study, and provided a signed consent form for participants and their parents/guardians to read and sign.

### Data analysis

Quantitative variables were shown as mean (*M*), standard deviation (*SD*) and frequency (%), respectively. All the findings were consistent when analyzed by time (pre- and post-test periods) and group (PI, and control groups) via repeated measures multivariate analysis of variance (MANOVA). The independent between-group variables included the two levels of PI and control groups. The independent within-group variables consisted of the two levels of pre- and post-test evaluation periods. The dependent variables were the depression, communication, trust and alienation variables. Homogeneity of variances and normality of data were evaluated by the levene and shapiro–wilk tests, respectively. The following categorical variables (age, grade and socio-economic status) were also analyzed by *x*^2^ test. Cohen’s *d* provided the small, medium, and large estimations of 0.02, 0.15, and 0.35 for the effect sizes, respectively [10]. All statistical analyses were performed in IBM SPSS Statistics, version 20.0 (IBM [27]. *p* values less than 0.05 were regarded as statistically significant.

## Results

### Sample characteristics

**Fig. 1:**
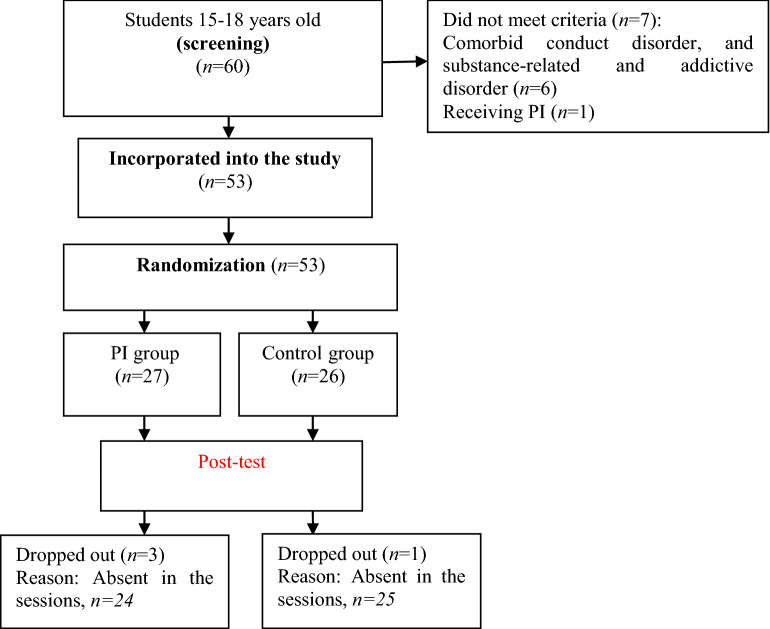
Distribution of study students

In this study, 60 high school girl students with moderate/mild depression were recruited and assessed for eligibility (Fig. 1). Of these, 4, 2, and 1 students were excluded due to having conduct and addictive disorders as related by their parents and peers and receiving PI before entering the study, respectively. The remaining 53 students were randomly into the two mentioned groups. 3 and 1 students in the PI and control groups dropped out during the PI post-test, respectively. Finally, 49 students (*n *= 24 and 25 for the PI and control groups, respectively) completed the BDI-II and IT-SR without missing variables.

As shown in Table 2, the total sample included 49 female students aged 17.15 ± 3.28 and 16.84 ± 3.17 years in the PI and control groups, respectively. A summary of the students’ demographic data is presented in Table 2. The PI and control groups showed no statistically significant differences in terms of the demographic variables of age, grade, and socio-economic status (*p *< 0.05; Table 2).

**Table 2:** Participant demographics

	PI group (*n *= 24)	Control group (*n *= 25)	*p*
	*M *± *SD*	*M *± *SD*	
Age, years	17.15 ± 3.28	16.84 ± 3.17	0.595
Grade			0.366
First grade of high school	15.54 ± 1.08	15.01 ± 0.96	
Second grade of high school	16.89 ± 0.89	16.25 ± 1.14	
Third grade of high school	17.26 ± 1.78	17.47 ± 1.33	
Fourth grade of high school	18.91 ± 1.38	18.63 ± 1.27	
Socio-economic status			0.771
Low, *n* (%)	11 (45.8)	12 (46.2)	
Medium, *n* (%)	10 (41.7)	6 (23.1)	
High, *n* (%)	3 (12.5)	7 (30.7)	

*M* mean, *SD* standard deviation, p values refer to the χ^2^ test

### Descriptive findings

Table 3 presents a comparison of the means and standard deviations of the BDI-II and IT-SR variables between the two groups at the pre-test and the post-test.

**Table 3:** Mean and standard deviation of the BDI-II and IT-SR variables at the pre-test and the post-test between the two groups

Variable	Groups	*M *± *SD* at the baseline	*M *± *SD* at the 2 month
Depression	PI	18.23 ± 7.20	13.34 ± 7.00
	Control	18.06 ± 8.35	18.23 ± 8.49
Communication	PI	22.43 ± 4.48	26.01 ± 4.69
	Control	24.26 ± 4.27	23.93 ± 4.05
Trust	PI	14.13 ± 3.31	16. 16 ± 3.48
	Control	15.80 ± 2.72	15.66 ± 2.53
Alienation	PI	11.90 ± 2.45	8.10 ± 2.42
	Control	11.16 ± 2.59	11.15 ± 2.66

*M*: mean, *SD*: standard deviation, *TSR* teacher–student relationship, *PI* positive intervention

*N *= 49

There was an improvement in the variables of communication and trust and a decrease in alienation and depression from the pre-test to the post-test stages for the PI, but not for the control group.

### Inferential findings

The homogeneity of variances and normality of data were evaluated by the levene and shapiro–wilk tests, respectively (*p *> 0.05). Table 4 shows a comparison of the BDI-II and IT-SR variables at the pre-test and the post-test.

**Table 4:** Main and interaction effects comparison of the BDI-II and IT-SR measure between the two groups

	Main effect						Interaction effect		
	Within-group			Between-group					
	*F*	*P*	*d*	*F*	*P*	*d*	*F*	*p*	*d*
Depression	25.43	0.001	0.37	9.54	0.005	0.11	12.22	0.001	0.15
Communication	11.95	0.007	0.14	8.56	0.001	0.10	14.53	0.001	0.17
Trust	15.44	0.001	0.18	8.10	0.001	0.11	12.47	0.001	0.14
Alienation	10.94	0.030	0.12	8.03	0.001	0.12	10.60	0.012	0.11

*F* and *p* values refer to repeated-measures MANOVA comparing groups, *d *= Cohen’s effect size

TSR variables and depression in the IT-SR and BDI-II were evaluated for the two groups at the baseline and 2 months after beginning the investigation. Repeated measures MANOVA depicted significant multivariable effects for the groups (*F*_=_1.17, *p *= 0.001, *d *= 0.05, *V *= 0.059), and evaluation periods (*F *= 8.02, *p *= 0.001, *d *= 0.30, *V *= 0.301), as well as significant interaction effects between the groups and evaluation periods (*F *= 9.80, *p *= 0.001, *d *= 0.34, *V *= 0.344).

The univariable between-group analysis displayed significant effects for communication, and trust, with higher scores of the PI compared to the control group. Also, alienation, and depression, were assessed with the lower score of the same group (Table 4). Although, these effect sizes are small, but they are indicating the PI positive effect on this research variables.

The univariable within-group analysis demonstrated that depression, communication, trust, and alienation had significantly improved from the pre-test to the post-test stages regardless of the groups. Small to medium effect sizes were also attained and large effect size was obtained for depression (Table 4). This recommends that depressed students have significantly improved the discussed variables, regardless of the groups.

In addition, there were significant interaction effects between the groups and evaluation periods in terms of depression, communication, trust, and alienation. Small to medium effect sizes were observed (Table 4), which showed that the PI group depression, communication, trust, and alienation scores were significantly better in comparison with the control group at the pre-test, therefore there was a significant difference between the groups by considering the post-test, and also depression, communication, trust, and alienation scores of the PI group were significantly improved at the pre-test.

Post-hoc comparisons using the Bonferroni’s test were indicative of significant effects for the depression, communication, trust, and alienation associated with the higher scores of the PI compared to the control group (*p *< 0.001). It is noteworthy to state that the above mentioned variables scores in the PI group had greater improvements in comparison with the control group.

## Discussion

This study followed the objective of determining PI efficacy in the depression and TSR of Iranian high school students with depression through IT-SR. The results revealed that PI was able to exert effective changes on IT-SR, i.e., increased communication and trust and decreased alienation in IT-SR after PI training (*p *< 0.05; Table 4).

The literature review showed that this study was the only investigation dealing with PI effects on communication, trust, and alienation among high school students. Notwithstanding, consistent with previous studies, there is a report that show positive group psychotherapy helped improve self-esteem, interpersonal relationships, and communication skills in 33 students [32]. The content of the program was partly similar with this PI. The contents include emotional expression, finding merits, self-disclosure and sharing memories about family members, one’s past, present and future [32]. Although, PI sessions does not directly address TSR and this is a positive side effect of the skills that were taught, but it seems that Lee measures communication skills and PI program, described in Table 1, would impact the teacher student relationship. Its confirmation will require further research.

Additionally, our results showed that the 8-week PI training significantly alleviated depression symptoms among high school students with depression, which is consistent with other studies [22, 50, 52]. A meta-analysis of school-based intervention programs targeted at reducing symptoms of posttraumatic stress disorder and depression suggest that intervention provided within the school setting can be effective in helping students following traumatic events and depression symptoms. Only 8 out of the 19 studies that entered the meta-analyses used art therapy, narrative therapy, cognitive–behavioral therapy, eye movement desensitization and reprocessing, and meditative & bioenergetics exercises [47]. The overall effect size for the 8 studies was *d *= 0.54, indicating a large effect in relation to reducing depression symptoms, which is consistent with our findings (*d *= 0.37).

Regarding results in pervious studies and obtained results in present research, it would be mention that PI effectiveness on depression appears to be associated with the mechanisms of change described by Lyubomirsky and Layous. These researchers suggested that positive psychotherapy interventions due to increased positive emotions, behaviors, thoughts, satisfaction, psychological needs such as autonomy, love and belonging and communication boost the people’s mood [37]. Further research is needed to prove any change mechanisms association with PI sessions though similar change processes can be hypothesized to have involved our study population.

### Limitations of the study

This study had the limitation of being performed only on high school students with a small sample size and hence, the intervention could not be generalized to other samples like elementary school students. Future studies should be conducted with larger sample sizes among various participants regarding educational levels. Participants of the study are only girls. Gender effects have not been reported for the success of PI so far. However, repeating and extending these findings with more wide-ranging samples seems promising. Depression and TSR were evaluated by BDI-II and IT-SR, respectively. Also, only self-reports used (no parent/caregiver reports or teacher report measures). Using only one measure to evaluate each variable and ignoring confounding variables in intervention designs may lead results to not representing the context of the intervention and solo effect of the intervention program. Some other limitations included the lack of conducting a follow-up plan and ruling out the therapist effects or non-specific factors of group psychotherapy. Therefore, future studies should consider the issues mentioned.

## Conclusion

In this study, the small to medium effect sizes obtained in the repeated measures MANOVA for the main effect of the through IT-SR, while large effect size was attained for depression through BDI-II measures. The average effect size was *F*^*2*^= 0.18, which is considered a medium effect size. Indeed, most of the effect sizes are smaller than that reported in meta-analysis results for PI [54]. In terms of clinically significance, it indicates a greater difference between the PI and control groups, yet the sample sizes in this research were smaller, which would lead to a lower statistical power in comparison with the study accomplished by Sin and Lyubomirsky [54].

In this investigation, we attempted to enhance TSR and decrease depression symptoms with 8-week PI program. As a result, PI efficacy in improving communication and trust and reducing alienation and depression among high school students with mild-moderate depressive symptoms was documented. Therefore, this intervention could function as an unspecific component of a stepped care approach for teenage girls suffering from depression. It’s hard to say whether there were other factors at play, so at this point this is promising information but not conclusive. There may be other issues not accounted for, such as school climate, selectivity of the sample, etc. Note that these interventions are not going to replace current treatment techniques for students, and they may serve supplementary purposes. As a pilot study, it is expected that our findings could help future research to design better PI for depression symptoms and TSR in high school.

## Data Availability

All data and material are available at the Department of Education and Psychology at the Yasouj University.

## References

[1] American Psychiatric Association (2013). DSM 5.

[2] An DY, Ahn GYR (2012). The effect of self-esteem improvement program for middle school students. J Korean Assoc Psychother.

[3] Armsden GC, Greenberg MT (1987). The Inventory of Parent and Peer Attachment: individual differences and their relationship to psychological well-being in adolescence. J Youth Adolesc.

[4] Beck AT, Steer R, Brown GK (1996). Beck depression inventory II: manual.

[5] Bolier L, Haverman M, Westerhof GJ, Riper H, Smit F, Bohlmeijer E (2013). Positive psychology interventions: a meta-analysis of randomized controlled studies. BMC Public Health..

[6] Bonhauser M, Fernandez G, P€uschel K (2005). Improving physical fitness and emotional well-being in adolescents of low socioeconomic status in Chile: results of a school-based controlled trial. Health Promot Int.

[7] Brinkwortha ME, McIntyrea J, Jurascheka AD, Gehlbach H (2018). Teacher-student relationships: the positives and negatives of assessing both perspectives. J Appl Dev Psychol.

[8] Chakhssi F, Kraiss JT, Sommers-Spijkerman M, Bohlmeijer ET (2018). The effect of positive psychology interventions on well-being and distress in clinical samples with psychiatric or somatic disorders: a systematic review and meta-analysis. BMC Psychiatry..

[9] Chan ES, Koh D, Teo YC, Hi Tamin R, Lim A, Fredericks S (2013). Biochemical and psychometric evaluation of self-healing qigong as a stress reduction tool among first year nursing and midwifery students. Compl Ther Clin Pract.

[10] Cohen J (1992). A power primer. Psychol Bull.

[11] Cohn MA, Fredrickson BL, Brown SL, Mikels JA, Conway AM (2009). Happiness unpacked: Positive emotions increase life satisfaction by building resilience. Emotion..

[12] Critchley H, Gibbs S (2012). The effects of positive psychology on theefficacy beliefs of school staff. Educ Child Psychol.

[13] Csikszentmihalyi M (1990). Flow: the psychology of optimal experience.

[14] Curtin SC, Warner M, Hedegaard H (2016). Increase in suicide in the United States, 1999–2014. NCHS Data Brief..

[15] D’raven LL, Pasha-Zaidi N (2014). Positive psychology interventions: a review for counselling practitioners interventions. Can J Counsel Psychother.

[16] Dias M, Vellarde GC, Olei B, Teofilo SAE, De-Barros RI (2014). Effects of electroacupuncture on stress-related symptoms in medical students: a randomized placebo-controlled study. Acupunct Med.

[17] Dockray S, Steptoe A (2010). Positive affect and psychobiological processes. Neurosci Biobehav Rev.

[18] Emami H, Ghazinour M, Rezaeishiraz H, Richter J (2007). Mental health of adolescents in Tehran, Iran. J Adolesc Health.

[19] Fredrickson BL, Branigan C (2005). Positive emotions broaden the scope of attention and thought-actionrepertoires. Cogn Emot.

[20] Gehlbach H, Brinkworth M, Harris A (2012). Changes in teacher-student relationships. Br J Educ Psychol.

[21] Ghofranipour F, Saffari M, Mahmoudi M, Montazeri A (2013). Demographical and psychological determinants of depression, among a sample of Iranian male adolescents. Int J Prev Med..

[22] Guo Y-F, Zhang X, Plummer V, Lam L, Cross W, Zhang J-P (2017). Positive psychotherapy for depression and self-efficacy in undergraduate nursing students: a randomized, controlled trial. Int J Ment Health Nurs.

[23] Hendriks T, Schotanus-Dijkstra M, Hassankhan A, Graafsma T, Bohlmeijer E, de Jong J (2018). The efficacy of positive psychology interventions from non-Western countries: a systematic review and meta-analysis. Int J Wellbeing..

[24] Hill RM, Yaroslavsky I, Pettit JW (2015). Enhancing depression screening to identify college students at risk for persistent depression symptoms. J Affect Disord.

[25] Hisli Şahin N, Durak Batıgün A, Koç V (2011). The relationship between depression, and interpersonal style, self-perception, and anger. Turk Psikiyatri Derg..

[26] Horowitz JL, Garber J, Carber JA, Young JF, Mufson L (2007). Prevention of depressive symptoms in adolescents: a randomized trial of cognitive–behavioral and interpersonal prevention programs. J Consult Clin Psychol.

[27] IBM Corp (2011). IBM SPSS statistics for windows, version 20.0.

[28] Juvonen J, Alexander PA, Winne PH (2006). Sense of belonging, social bonds, and school functioning. Handbook of educational psychology.

[29] Lambert NM, Fincham FD, Stillman TF (2012). Gratitude and depressive symptoms: the role of positive reframing and positive emotion. Cogn Emot.

[30] Lee Duckworth A, Steen TA, Seligman ME (2005). Positive psychology in clinical practice. Annu Rev Clin Psychol.

[31] Lee EH, Lee SJ, Hwang ST, Hong SH, Kim JH (2017). Reliability and validity of the beck depression inventory-II among Korean adolescents. Psychiatry Investig..

[32] Lee EJ (2015). The effect of positive group psychotherapy on self-esteem and state anger among adolescents at Korean immigrant churches. Arch Psychiatr Nurs.

[33] Lemmens LH, van Bronswijk SC, Peeters F, Arntz A, Hollon SD, Huibers MJ (2019). Long-term outcomes of acute treatment with cognitive therapy v. interpersonal psychotherapy for adult depression: follow-up of a randomized controlled trial. Psychol Med.

[34] Lever TB, Strauss C, Cavanaqh K, Jones F (2014). The effectiveness of self-help mindfulness-based cognitive therapy in a student sample: a randomized controlled trial. Behav Res Ther.

[35] Longobardi C, Prino LE, Marengo D, Settanni M (2016). Student-teacher relationships as a protective factor for school adjustment during the transition from middle to high school. Front Psychol..

[36] Lyubomirsky S, King LA, Diener E (2005). The benefits of frequent positive affect. Psychol Bull.

[37] Lyubomirsky S, Layous K (2013). How do simple positive activities increase well-being?. Curr Dir Psychol Sci.

[38] Madadi Zavareh S, Golparvar M, Aghaie A (2018). Effect of positive existential therapy and positive Iranian–Islamic therapy on depression and anxiety of female students with social anxiety. Islamic Life Style..

[39] Majd E, Talepasand S, Rezaei MA (2017). A structural model of depression based on interpersonal relationships: the mediating role of coping strategies and loneliness. Noro Psikiyatr Ars..

[40] Modabber-Nia MJ, Shodjai-Tehrani H, Moosavi SR, Jahanbakhsh-Asli N, Fallahi M (2007). The prevalence of depression among high school and preuniversity adolescents: rasht, northern Iran. Arch Iran Med..

[41] Murray C, Zvoch K (2011). Teacher–student relationships among behaviorally at-risk African American youth from low-income backgrounds: student perceptions, teacher perceptions, and socioemotional adjustment correlates. J Emot Behav Disord.

[42] Murray C, Zvoch K. The Inventory of Teacher–Student Relationships: factor structure, reliability, and validity among African American youth in low-income urban schools. Paper presented at the annual meeting of the American Educational Research Association, San Diego, CA; 2009.

[43] Pietrowsky R, Mikutta J (2012). Effects of positive psychology interventions in depressive patients: a randomized control study. Psychology..

[44] Rashid T (2014). psychotherapy: a strength-based approach. J Posit Psychol.

[45] Roeser RW, Galloway MK, Pajares F, Urban T (2002). Studying motivation to learn during early adolescence: a holistic perspective. Academic motivation of adolescents.

[46] Roorda DL, Koomen HMY, Spilt JL, Oort FJ (2011). The influence of affective teacher-student relationships on students’ school engagement and achievement: a meta-analytic approach. Rev Educ Res.

[47] Rolfsnes ES, Idsoe T (2011). School-based intervention programs for PTSD symptoms: a review and meta-analysis. J Trauma Stress.

[48] Saluja G, Iachan R, Scheidt PC, Overpeck MD, Sun W, Giedd JN (2004). Prevalence of and risk factors for depressive symptoms among young adolescents. Arch Pediatr Adolesc Med.

[49] Schrank B, Brownell T, Jakaite Z, Larkin C, Pesola F, Riches S (2016). Evaluation of a positive psychotherapy group intervention for people with psychosis: pilot randomised controlled trial. Epidemiol Psychiatr Sci.

[50] Schrank B, Riches S, Coggins T, Rashid T, Tylee A, Slade M (2014). WELLFOCUS PPT-modified positive psychotherapy to improve well-being in psychosis: study protocol; for feasibility of randomized controlled trial. Trials..

[51] Seligman MEP, Csikszentmihalyi M (2000). Positive psychology: an introduction. Am Psychol.

[52] Seligman MEP, Rashid T, Parks AC (2006). Positive psychotherapy. Am Psychol.

[53] Seligman MEP (2002). Authentic happiness: using the new positive psychology to realize your potential for lasting fulfillment.

[54] Sin NL, Lyubomirsky S (2009). Enhancing well-being and alleviating depressive symptoms with positive psychology interventions: a practice-friendly meta-analysis. J Clin Psychol.

[55] Skinner E, Greene T, Good TL (2008). Perceived control, coping, and engagement. 21st century education: a reference handbook.

[56] Spilt JL, Koomen HMY, Thijs JT (2011). Teacher well being: the importance of teacher–student relationships. Educ Psychol Rev.

[57] Suldo MS, McMahan MM, Chappel AM, Bateman LP (2014). Evaluation of the teacher–student relationship inventory in American high school students. J Psychoeduc Assess.

[58] Sweeting H, West P (2003). Sex differences in health at ages 11, 13 and 15. Soc Sci Med.

[59] Ziaei R, Viitasara E, Soares J, Sadeghi-Bazarghani H, Dastgiri S, Zeinalzadeh AH, Bahadori F, Mohammadi R (2017). Suicidal ideation and its correlates among high school students in Iran: a cross-sectional study. BMC Psychiatry..

